# Supplementary material for: Reversibly Migratable Fluorescent Probe for Precise and Dynamic Evaluation of Cell Mitochondrial Membrane Potentials
Source: Biosensors (Basel). 2022 Sep 27;12(10):798. doi: 10.3390/bios12100798 (PMC9599583; doi:10.3390/bios12100798)
Supplement: Supplementary file 1 [file biosensors-12-00798-s001.zip › biosensors-1914019-supplementary.pdf]

biosensors

Supplementary Materials

Reversibly Migratable Fluorescent Probe for Precise and Dynamic Evaluation of Cell Mitochondrial Membrane Potentials

Guofen Song [1], Haiwei He [1,2], Wanling Chen [1], Yuanliang Lv [1], Paul K. Chu [3,4,5], Huaiyu Wang [1,2,*] and Penghui Li [1,2,*]

[1] Institute of Biomedicine and Biotechnology, Shenzhen Institute of Advanced Technology, Chinese Academy of Sciences, Shenzhen 518055, China
[2] University of Chinese Academy of Sciences, Beijing 100049, China
[3] Department of Physics, City University of Hong Kong, Tat Chee Avenue, Kowloon, Hong Kong 999077, China
[4] Department of Materials Science and Engineering, City University of Hong Kong, Tat Chee Avenue, Kowloon, Hong Kong 999077, China
[5] Department of Biomedical Engineering, City University of Hong Kong, Tat Chee Avenue, Kowloon, Hong Kong 999077, China
* Correspondence: hy.wang1@siat.ac.cn (H.W.); ph.li@siat.ac.cn (P.L.); Tel.: +86-755-86392211 (P.L.)

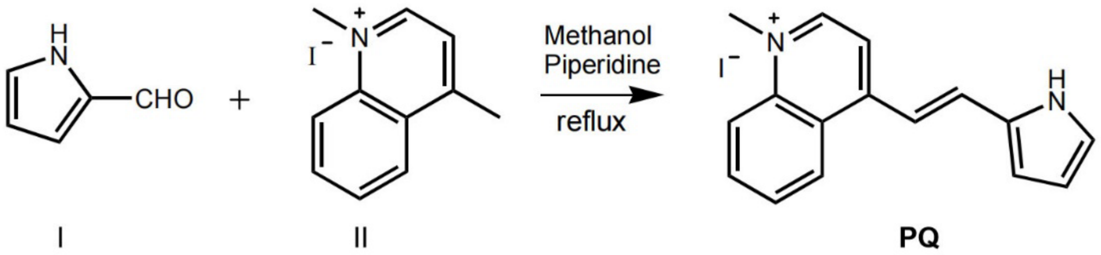

Scheme S1. Synthetic route of **PQ**.

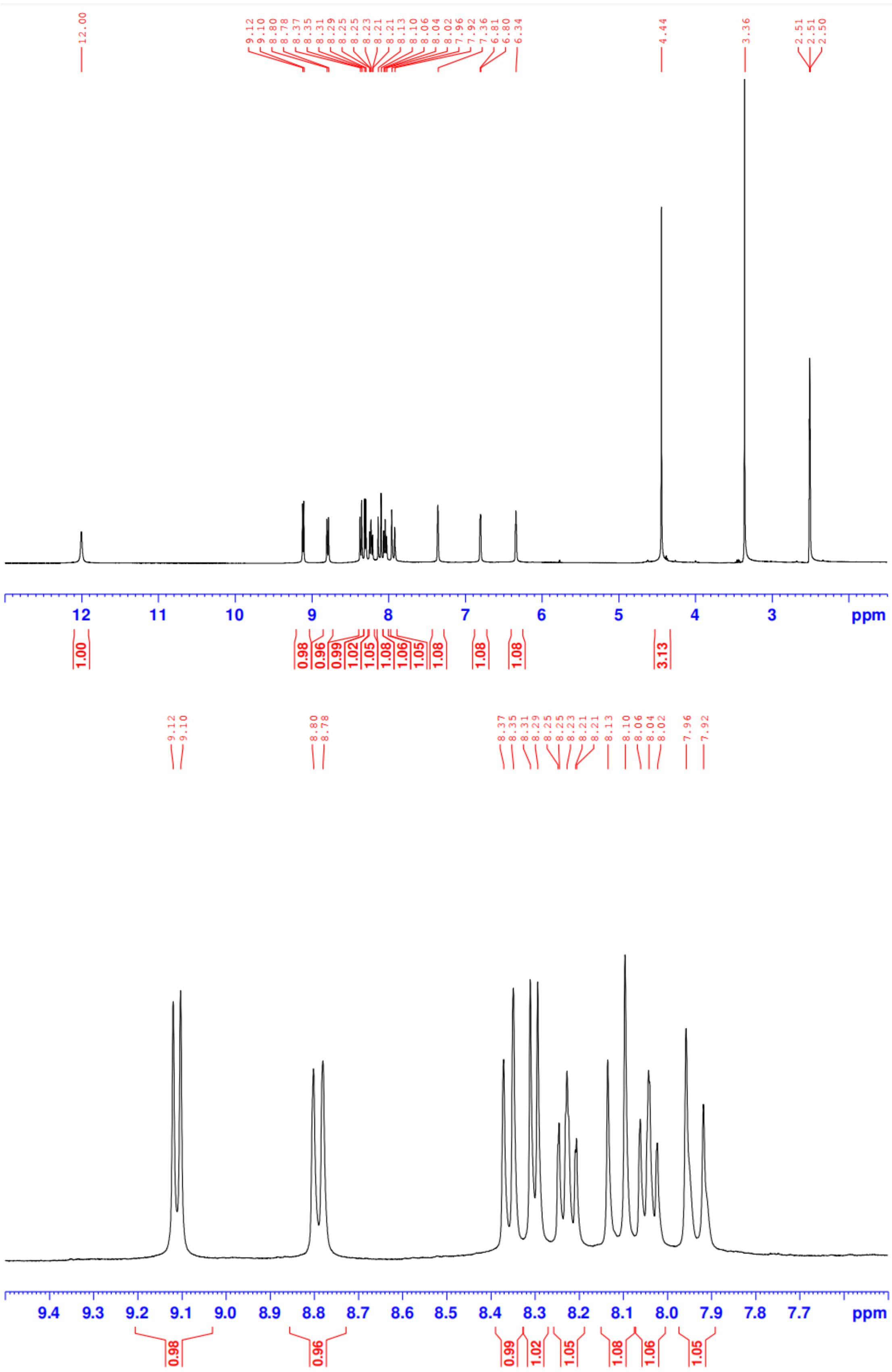

Figure S1. ^{1}H NMR spectrum of **PQ**.

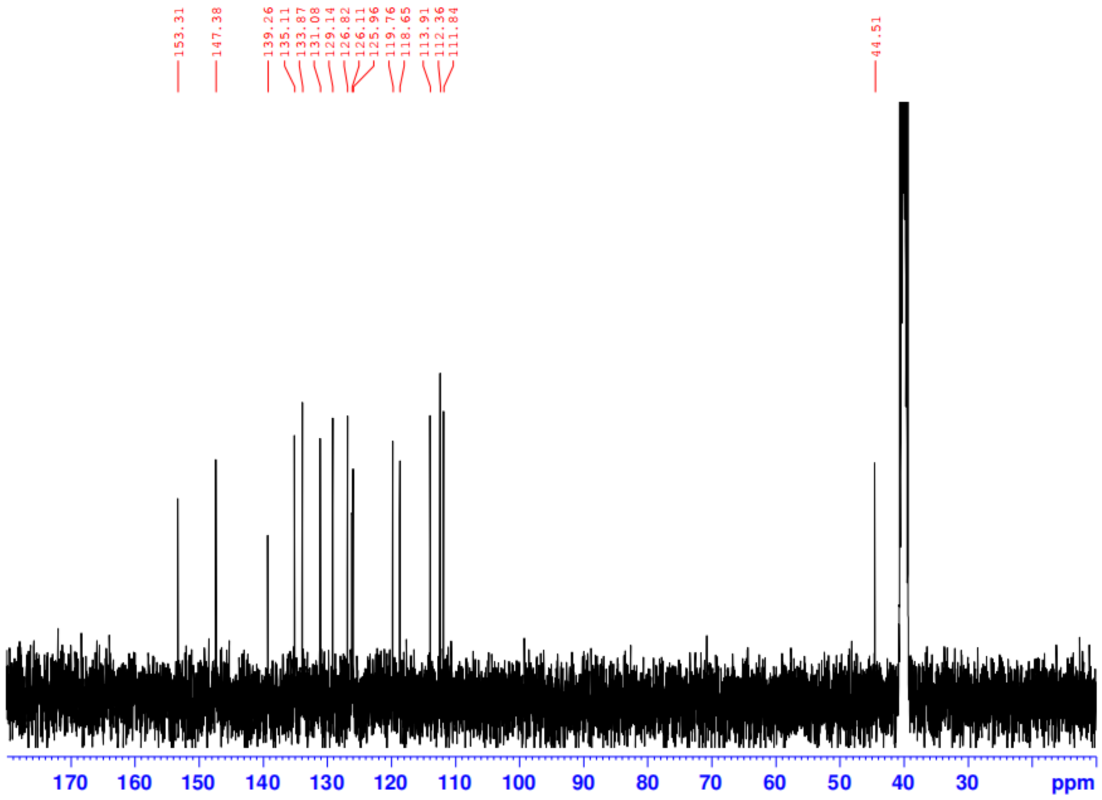

Figure S2. ^{13}C NMR spectrum of **PQ**.

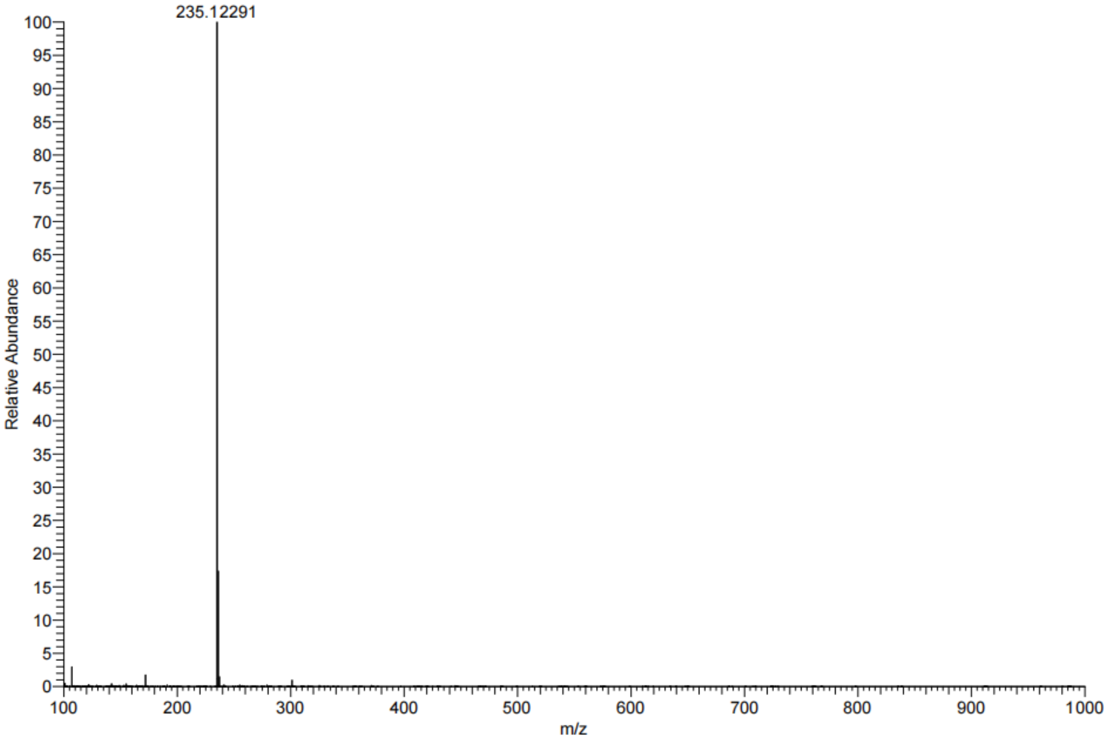

Figure S3. HRMS spectrum of **PQ**.

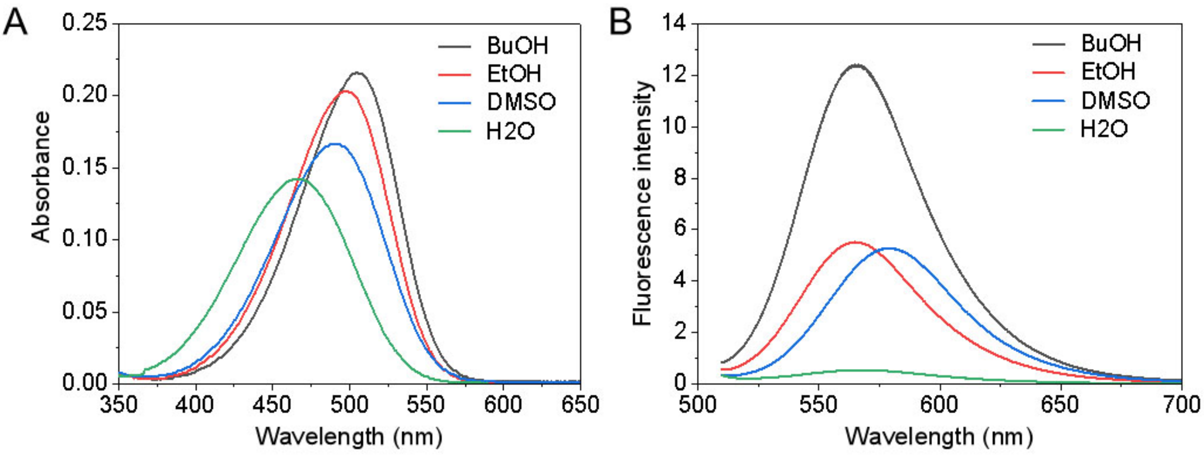

Figure S4. UV-vis absorption (**A**) and fluorescence spectra (**B**) of 5 μM **PQ** in various solvents with different polarities.

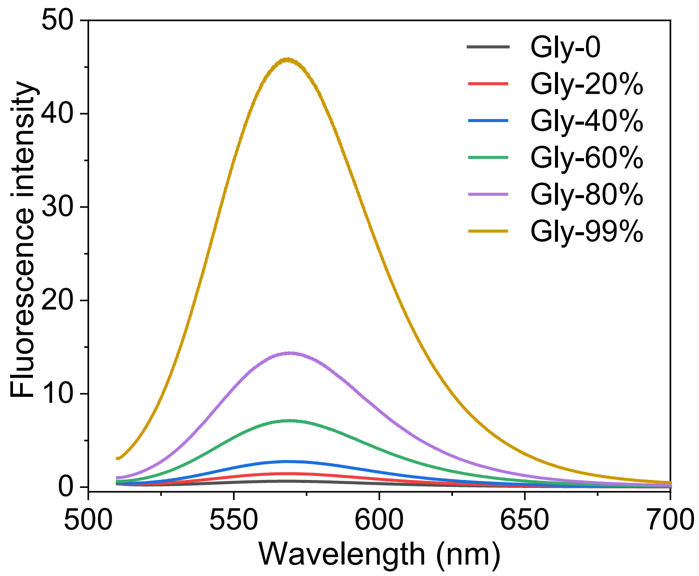

Figure S5. Fluorescence spectra of 5 μM **PQ** in H_2O/glycerol mixtures with various glycerol fractions.

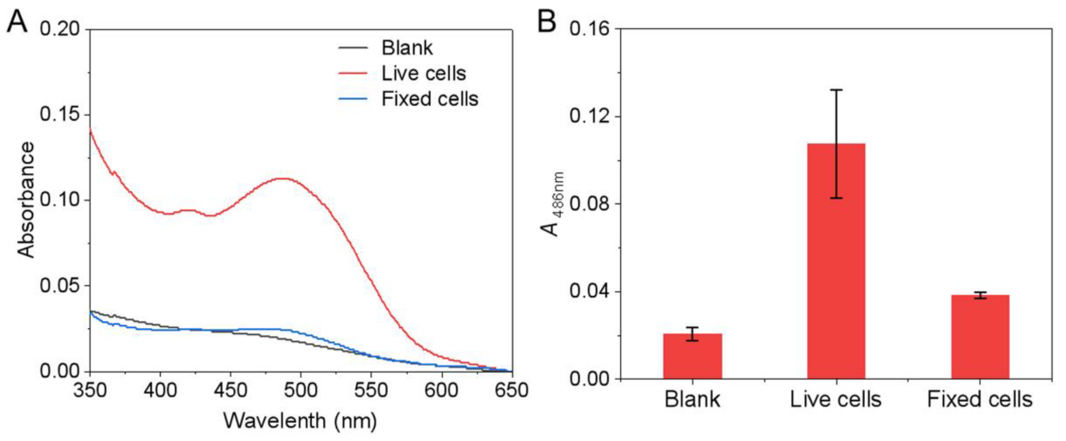

Figure S6. The demonstration for **PQ** translocation. (**A**) The absorbance spectra of lysates of the mitochondria isolated from cells stained with **PQ** before and after fixation. (**B**) Comparison of the absorbance at 486 nm, data represent mean±SD (n = 3).

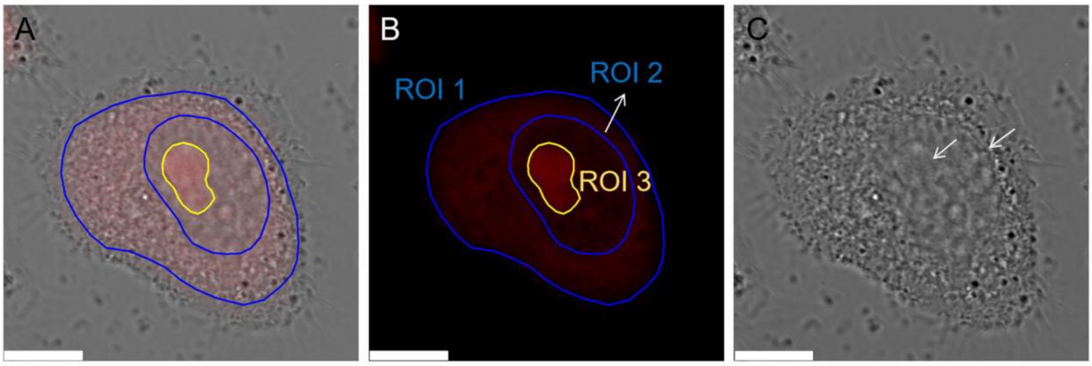

Figure S7. CLSM images of HeLa cells stained by 10 μM **PQ** after treatment of 100 μM CCCP for 10 min. (**A**) Merge of Fluorescent and bright field images; (**B**) Fluorescent image; (**C**) Bright field image. λ_{ex}: 488 nm, λ_{em}: 550–650 nm, scale bar: 10 μm. The cell membrane, nucleus and nucleolus were distinct. And the whole cell inside the cell membrane, the nuclear and the nucleolus were sketched by the big blue circle (ROI 1), the small blue circle (ROI 2) and the yellow circle (ROI 3) individually.

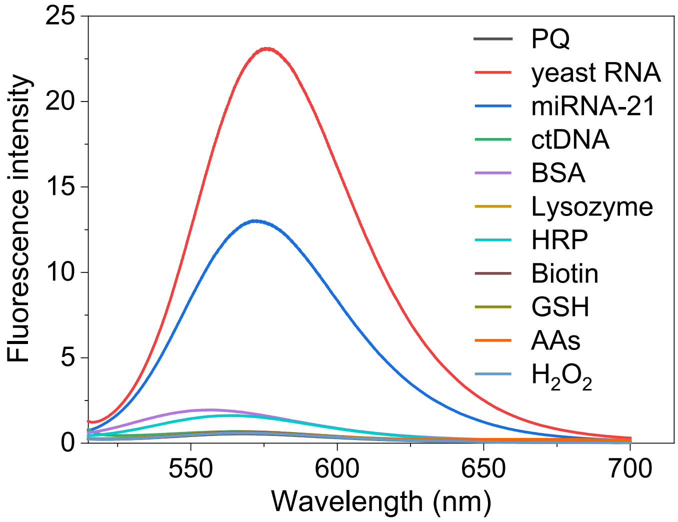

Figure S8. Fluorescence spectra of 5 μM **PQ** in the presence of *yeast* RNA, miRNA-21, *ct*DNA, proteins, biotin, GSH, amino acids and H_2O_2 in PBS. The proteins include BSA, Lyso and HRP. The amino acids (AAs) include Hcy, Cys, Gly, Ala, Nor, Glu, His, Iso, Leu, Met, Phe, Pro, Ser, Thr, Try, and Tyr.

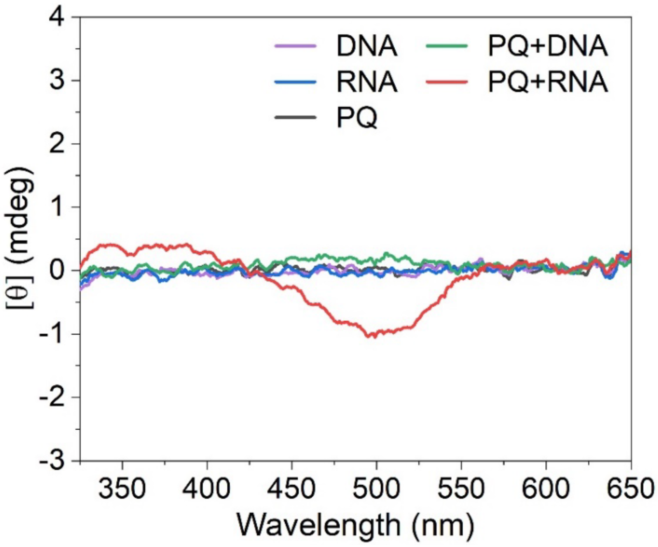

Figure S9. CD spectra of 20 μM **PQ** with or without DNA/RNA (0.5mg/mL) in PBS.

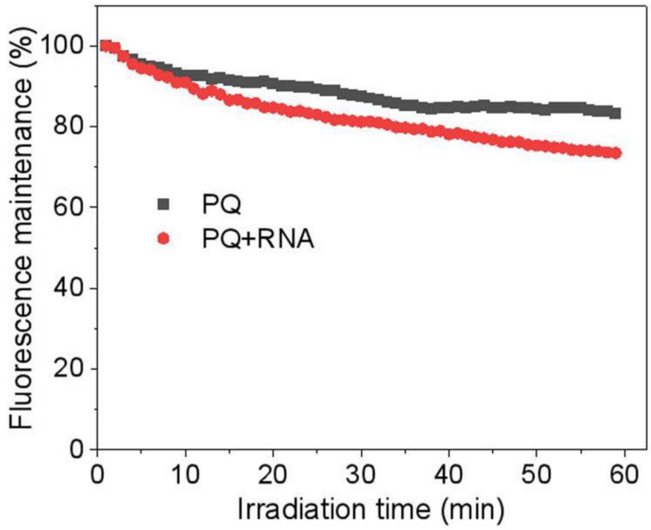

Figure S10. The fluorescence stability of 5 μM **PQ** in solution in absence and presence of RNA with light irradiation time. λ_{ex} = 480 nm, excitation slit: 10, λ_{em} = 575 nm.

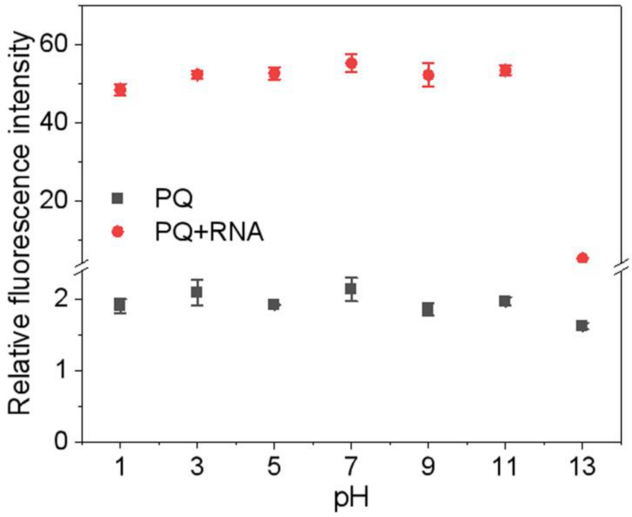

Figure S11. The fluorescence intensity changes of **PQ** (5 μM) at 575 nm in absence and presence of RNA under various pH. .

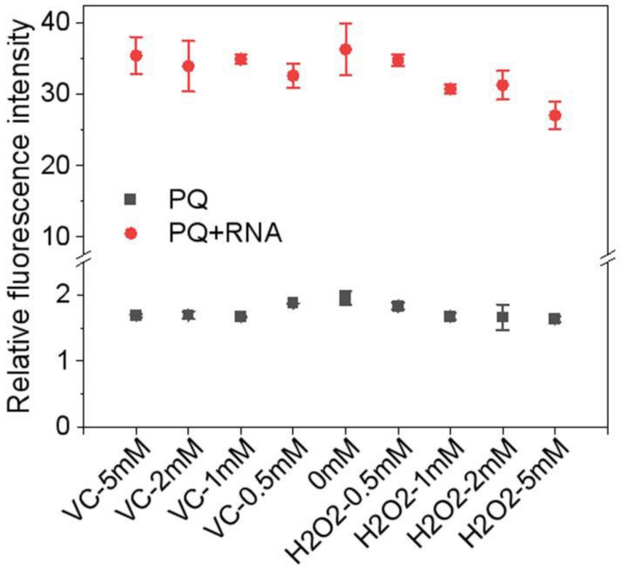

Figure S12. The fluorescence intensity changes of **PQ** (5 μM) at 575 nm in presence of oxidizing agent (0.5–5 mM H_2O_2) and reducing agent (0.5–5 mM VC).

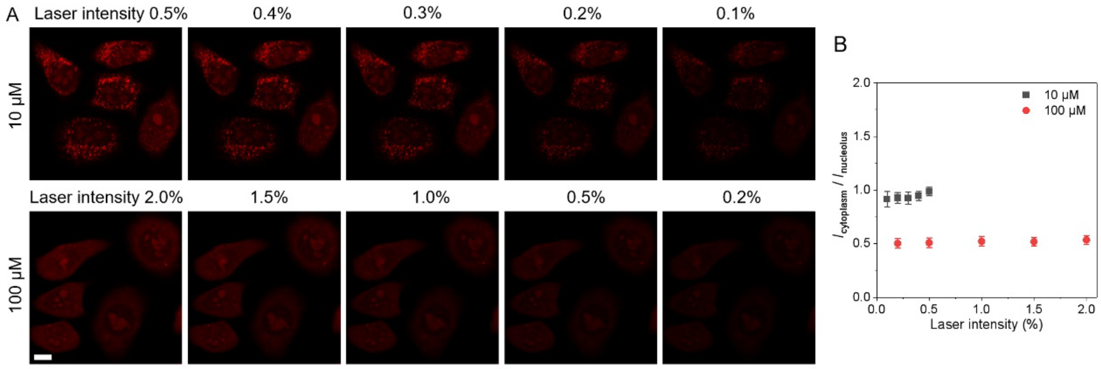

Figure S13. Resistance to imaging parameters. (**A**) The CLSM images of **PQ**-stained live HeLa cells after treatment of 10 μM and 100 μM CCCP excited by a series of laser intensities (0.1%–2.0%). The images of cells in 10 μM CCCP were obtained excited by laser intensities 0.1%–0.5% because the fluorescence was saturated under laser intensity ≥ 0.6%. (**B**) Corresponding $I_{cytoplasm}$ / $I_{nucleolus}$, data represent mean ± SD (n = 5).

Table S1. The photophysical properties of **PQ**.

Solvent	λ_{Ab} /nm	λ_{em} /nm	Stokes shift /nm	ε /M^{-1} •cm^{-1}	Φ_{FL} /%
n-BuOH	505	566	61	4.3×10^4	2.2
EtOH	498	565	67	4.1×10^4	0.88
DMSO	490	579	89	3.3×10^4	1.1
Water	465	567	102	2.8×10^4	0.13
+RNA [a]	504	576	72	2.7×10^4	5.6
+DNA [a]	463	567	104	3.4×10^4	0.14
Glycerol	494	569	75	2.8×10^4	12

λ_{Ab} and λ_{em} are the linear absorption and fluorescent maximum peaks, respectively, ε is molar absorptivity, and Φ_{FL} is fluorescence quantum yield using fluorescein (Φ = 0.95) as the standard.

[a] The value of DNA phosphate/compound and RNA phosphate/compound is 200.
